# A new *Oxyspirura* (Nematoda, Thelaziidae) in three captive non-human primate species

**DOI:** 10.3389/fvets.2025.1650452

**Published:** 2025-09-05

**Authors:** Ondřej Máca, David González-Solís

**Affiliations:** ^1^Department of Zoology and Fisheries, Faculty of Agrobiology, Food and Natural Resources, Czech University of Life Sciences Prague, Prague, Czechia; ^2^Department of Pathology and Parasitology, State Veterinary Institute Prague, Prague, Czechia; ^3^Department of Systematics and Aquatic Ecology, El Colegio de la Frontera Sur, Chetumal, Mexico

**Keywords:** Nematoda, *Oxyspirura*, primates, morphology, molecular analysis, eyes, phylogeny, new species

## Abstract

Members of *Oxyspirura* are mainly parasites in the eye of a wide variety of wild and domestic birds, as well as of some mammals. The latter group is represented by species found in non-human primates from zoological gardens. Recently, dead non-human primates of 12 species were examined to determine those with infections in the eyes, as well as their morphological and molecular identification. For that, 14 and six individual nematodes were used for the morphological and molecular analyses (*18S* rRNA, *28S* rRNA, ITS, and *cox1* genes), respectively. Three out of the 12 non-human primate species (*Leontopithecus chrysomelas, Saguinus midas*, and *Saguinus oedipus*) showed eye infection with nematodes, whose specific identification resulted in the erection of a new species, *Oxyspirura* (*Oxyspirura*) *tamarina* sp. nov. This species is characterized by having a divided buccal capsule, spicules unequal and dissimilar, gubernaculum present or absent, and variability in the number of precloacal papillae. All newly generated sequences were identical to each other. The new species differs morphologically from its congeners in the shape of the buccal capsule, length of spicules, and number and distribution of caudal papillae; molecularly, the genetic divergence was higher than 5% in all markers. Despite the morphological differences of the nematodes studied, the molecular analysis allowed us to recognize them as a sole species, thus becoming the third species of *Oxyspirura* reported in primates kept in captivity around the world.

## Introduction

The genus *Oxyspirura* Drasche in Stossich, 1898 includes around 89 known species, divided into several controversial and uncertain subgenera, according to the morphology of their buccal capsule, shape of spicules, and absence/presence of gubernaculum and cervical alae. However, the real number of valid species might be fewer since there exists a wide intraspecific variability in the diagnostic features (i.e., number and distribution of caudal papillae, presence/absence of gubernaculum), and many species were described based on a single or few specimens from the same host species. These parasites are heteroxenous nematodes usually found on the eye surface, under the nictitating membrane, as well as in the lacrimal ducts and other eye glands of a wide variety of wild and domestic birds and mammals. The adults of *Oxyspirura* deposit the eggs, which together with lacrimal secretions follow the tear ducts to the mouth where they are swallowed and eliminated through the feces. The eggs are ingested by cockroaches, crickets or grasshoppers that act as intermediate hosts (1, 2), while humans might get infected after the larvae penetrate skin and thus become a zoonosis (3).

Historically, most species of *Oxyspirura* have been found in birds, but there are two that were found in mammals (Primates) from zoological gardens, *O*. (*Oxyspirura*) *conjunctivialis* (von Linstow, 1907) Oliveira-Rodrigues et Freitas, 1964 in *Microcebus murinus* (Miller, 1777) and *Loris gracilis* Geoffrey, 1796 from Berlin Zoo, and *Nycticebus coucang coucang* (Boddaert, 1785) from Moscow Zoo, as well as *O*. (*O*.) *youngi* Addison, Forrester, Whitley et Curtis, 1986 in *Erythrocebus patas* Schreber, 1775 from Jacksonville Zoo, Florida (4–6). These nematode species were provisionally synonymized by Ivanova et al. (6) after the re-examination of the type specimens and failing to find significant morphological differences among specimens of both species, although there was a lack of molecular characterization and comparison among species.

Recently, non-human primates of 12 species and seven genera died in six zoological gardens and six private facilities in the Czech Republic. Some animals showed nematode infections in both eyes, which is why veterinarians and breeders sent them for examination after sudden death. Thus, the main goals of this study were to determine the non-human primate species with eye infections and to carry out the morphological and molecular identification of those nematodes.

## Methods

**Figure 1:**
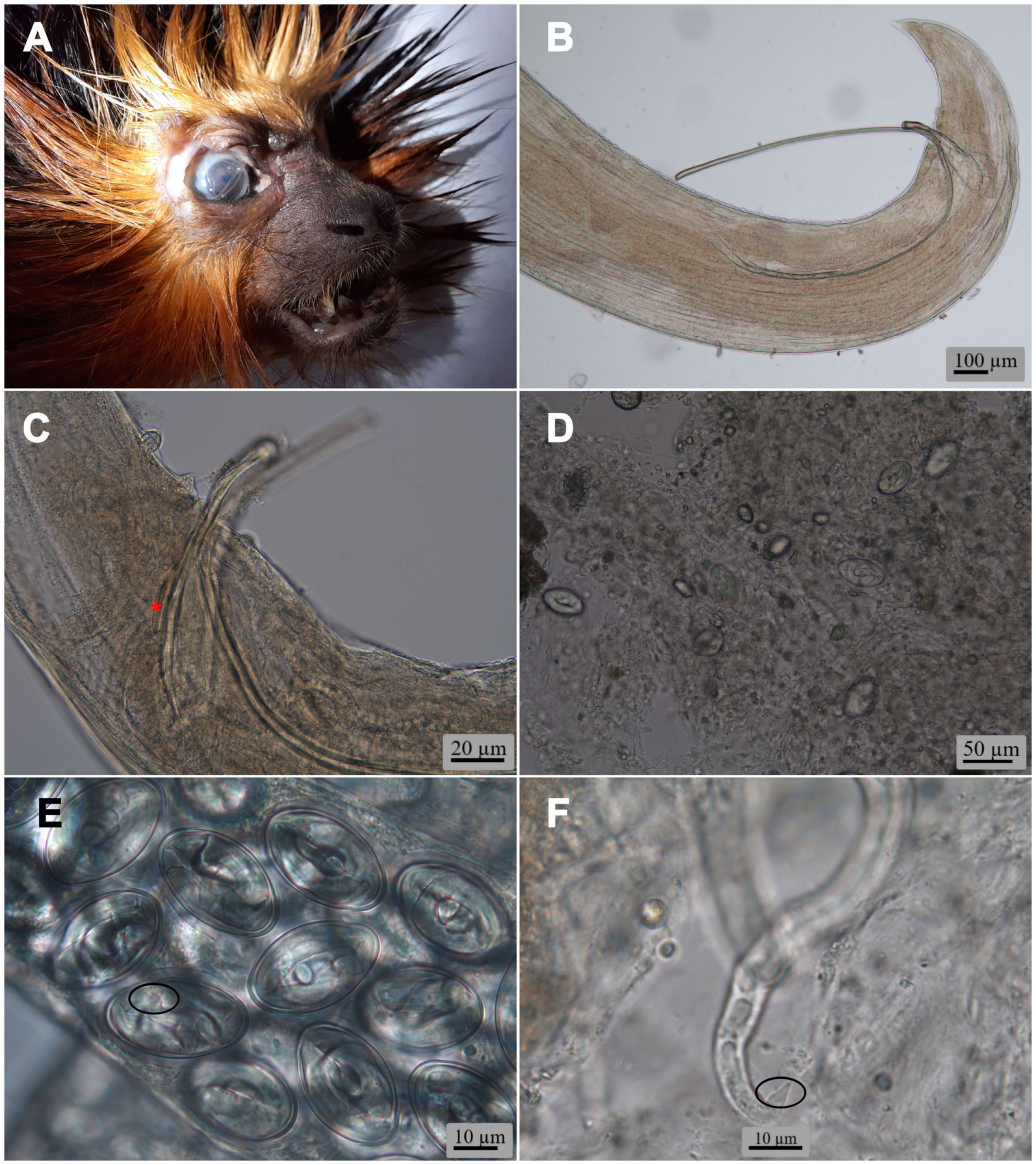
**(A)** Head of the golden-headed lion tamarin *Leontopithecus chrysomelas* (Kuhl, 1820) from a private facility in the Czech Republic, with nematodes in its right eye. *Oxyspirura* (*Oxyspirura*) *tamarina* sp. nov. light microscopy micrographs, **(B)** posterior end of male from *L*. *chrysomelas* showing both spicules and without gubernaculum; **(C)** region of cloaca of male from the cotton-top tamarin *Saguinus oedipus* (Linnaeus, 1758) showing right spicule, distal end of left spicule and gubernaculum (asterisk); **(D)** wet eye smear of *S*. *oedipus* from a Czech zoological garden, showing eggs; **(E)** eggs in uterus of female from *S*. *oedipus* (circle shows tail of larva); **(F)** larva manually released from egg (circle shows tail of larva) from the Midas tamarin *Saguinus midas* Linnaeus, 1758.

A total of 25 dead non-human primates, the common marmoset *Callithrix jacchus* (Linnaeus, 1758), the white-headed marmoset *Callithrix geoffroyi* (Humboldt, 1812), the eastern pygmy marmoset *Cebuella niveiventris* Lönnberg, 1940, the Colombian white-faced capuchin *Cebus capucinus* (Linnaeus, 1758), the golden lion tamarin *Leontopithecus rosalia* (Linnaeus, 1766), the Celebes crested macaque *Macaca nigra nigra* (Desmarest, 1822) (1 each); the western pygmy marmoset *Callithrix pygmaea* (Spix, 1823), the golden-headed lion tamarin *Leontopithecus chrysomelas* (Kuhl, 1820), the white-lipped tamarin *Saguinus labiatus* (Geoffroy in Humbolt, 1812), the Guianan squirrel monkey *Saimiri sciureus* (Linnaeus, 1758) (2 each); the cotton-top tamarin *Saginus oedipus* (Linnaeus, 1758) (3), and the Midas tamarin *Saguinus midas* Linnaeus, 1758 (8), from six zoological gardens and six private breeders were sent during 2019–2023 to the State Veterinary Institute Prague for necropsy. Nematodes in the orbital region were visible by naked eye (Figure 1A) and were collected by making a small cut on the eye surface and adding physiological saline. Additionally, to look for nematode eggs, wet smears of mucus around the eyes were taken from one eye of some animals (i.e., one of *L*. *rosalia, S*. *midas, S*. *oedipus*, two of *C*. *niveiventris*, and three of *S*. *labiatus*) from the same zoological garden, which were prepared by using saline solution and cotton wool. Some eggs were manually compressed to release the unhatched larvae. Nematodes were stored in 70% ethanol and 10% formalin for molecular and morphological identification, respectively, and cleared with a mixture of glycerin and water for physical examination. Those used for scanning electron microscopy (SEM) were dehydrated through an ethanol series, critical-point-dried, and sputter-coated with gold. Finally, they were examined using a scanning electron microscope (JEOL Model JSM6010, JEOL, Akishima, Tokyo, Japan) at El Colegio de la Frontera Sur (ECOSUR) Chetumal Unit. Type specimens were preserved in a mixture of ethanol-glycerin or on SEM stubs and deposited in the reference collection of ECOSUR, in Mexico (ECOPA). Drawings were made with the aid of an Olympus drawing tube attached to an Olympus CX31 microscope. Nematodes and eggs were observed and photographed by light microscopy with a Leica DM2500 LED optical microscope with digital camera Leica DMC5400, and microscope software Leica Application Suite X (Leica Microsystems, Wetzlar, Germany). Measurements are given in micrometers, unless otherwise indicated. The taxonomic classification of nematode follows that of Hodda (7).

For molecular analysis, one nematode from *L*. *chrysomelas*, two from *S*. *midas*, and three from *S*. *oedipus* were placed separately in 1.5 ml Eppendorf tubes containing 70% ethanol. Genomic DNA was extracted from each single nematode using the NucleoSpin tissue XS kit (Macherey-Nagel, Düren, Germany) according to the manufacturer's protocol and stored at −20 °C. Polymerase chain reactions (PCR) or nested PCR (nPCR) were used to amplify a fragment or partial sequence of *18S* rRNA, *28S* rRNA, *18S*-ITS1-*5.8S*-ITS2-*28S* region (ITS only hereafter), and *cox1* genes. PCR or nPCR were carried out in the final 25 μl volume containing 12.50 μl of GoTaq^®^ G2 Green Master Mix (Promega, Madison, Wisconsin, USA), 0.4 μM of each primer, 5 μl DNA template, and nuclease-free water. The sequences at these loci were amplified using the primer pairs shown in Table 1 and with recommended PCR annealing temperature based on the primer pairs used. The thermal cycler conditions were set at initial denaturation at 95 °C for 3 min; 30 cycles of amplification (95 °C for 30 s, 57–60 °C for 30 s, and 72 °C for 1 min) and ended with the final extension at 72 °C for 10 min. Negative (dH_2_O) controls were included in all the batches. The amplified PCR products were run on 1.0% (w/v) agarose gel with ethidium bromide stain. Subsequently, PCR products were purified with the help of ExoSAP-IT™ Express PCR Product Cleanup Reagent kit (Thermo Fisher Scientific, Waltham, MA, USA) according to the manufacturer's protocol and sent to Eurofins Genomics (Ebersberg, Germany) for direct sequencing using the amplification primers and sequenced in both forward and reverse.

**Table 1:** Primers used for the amplification of five DNA locus of the new nematode species.

**Target locus**	**Primer name**	**Sequence (5-3′)**	**Reference**
*18S* rRNA	Op18SF OP18SR OP18SIntF	CCGATTGATTCTGTCGGCGGTTA CACCTACGGAAACCTTGTTACGAC CTCAACACGGGAAAACTCACCTG	(21)
*28S* rRNA	28FWD 28REV	GGGAAAGAAGACCCTGTTGAG TTCTGACTTAGAGGCGTTCAG	(22)
ITS (*18S*-ITS1-*5.8S*-ITS2-*28S*)	PIR18 SPIR58 5FWD 5REV	TGAACCTGCGGAAGGATCATT GCAGCTRGCTGCGTKCTTCAT AGGTGAACCTGCGGAAGGATCATT TTCACGCCCTCTTGAACTCT	(6) (22)
*cox1*	OxyCOX1F OxyCOX1R Cox1NTF Cox1NTR Cox1NTInt	TGAGCTGGTTTAGGTGGTGCTA GAACCAGCTAACACAGGTACAGC TGATTGGTGGTTTTGGTAA ATAAGTACGAGTATCAATATC GGCTAGACAACTCTAAACG	(21) (23)

The newly generated sequences were compared with closely related spirurids found in the NCBI database (https://www.ncbi.nlm.nih.gov) by BLAST (https://blast.ncbi.nlm.nih.gov/Blast.cgi). Representative sequences for ITS region, *18S* rRNA and *cox1* genes were downloaded from GenBank and individual reference sequences were aligned using the online MAFFT version 7 (http://mafft.cbrc.jp/alignment/server/index.html) with default parameters. The best substitution model was selected in MEGA 12 software (version 12.0.11) (8). Phylogenetic trees were inferred based on the maximum likelihood method and the best-fit models. The evolutionary models Tamura 3 parameter model for *18S* rRNA and General Time Reversible model for *cox1* and ITS were chosen and the reliability of the trees was estimated by 1,000 bootstrap replications.

## Results

Only *L*. *chrysomelas, S*. *midas*, and *S*. *oedipus* out of the 12 dead non-human primate species showed eye infection with nematodes. Six individual parasites were collected in *L*. *chrysomelas* from a private breeder in 2022, 20 from *S*. *oedipus* and eight from *S*. *midas* from one zoological garden during 2019–2020. Only the eye smear of a still alive *S*. *oedipus* showed the presence of larvated eggs (Figure 1D), while the rest of wet smears were negative. Larvae inside the eggs and those manually freed were characterized by having a conical tail, pointed, without ornamentation (Figures 1E, F). The specific identification of the nematodes was as follows:

## Taxonomic summary

Family Thelaziidae Railliet, 1910

Genus *Oxyspirura* Drasche in Stossich, 1898

Subgenus *Oxyspirura* Skrjabin, 1931

**Figure 2:**
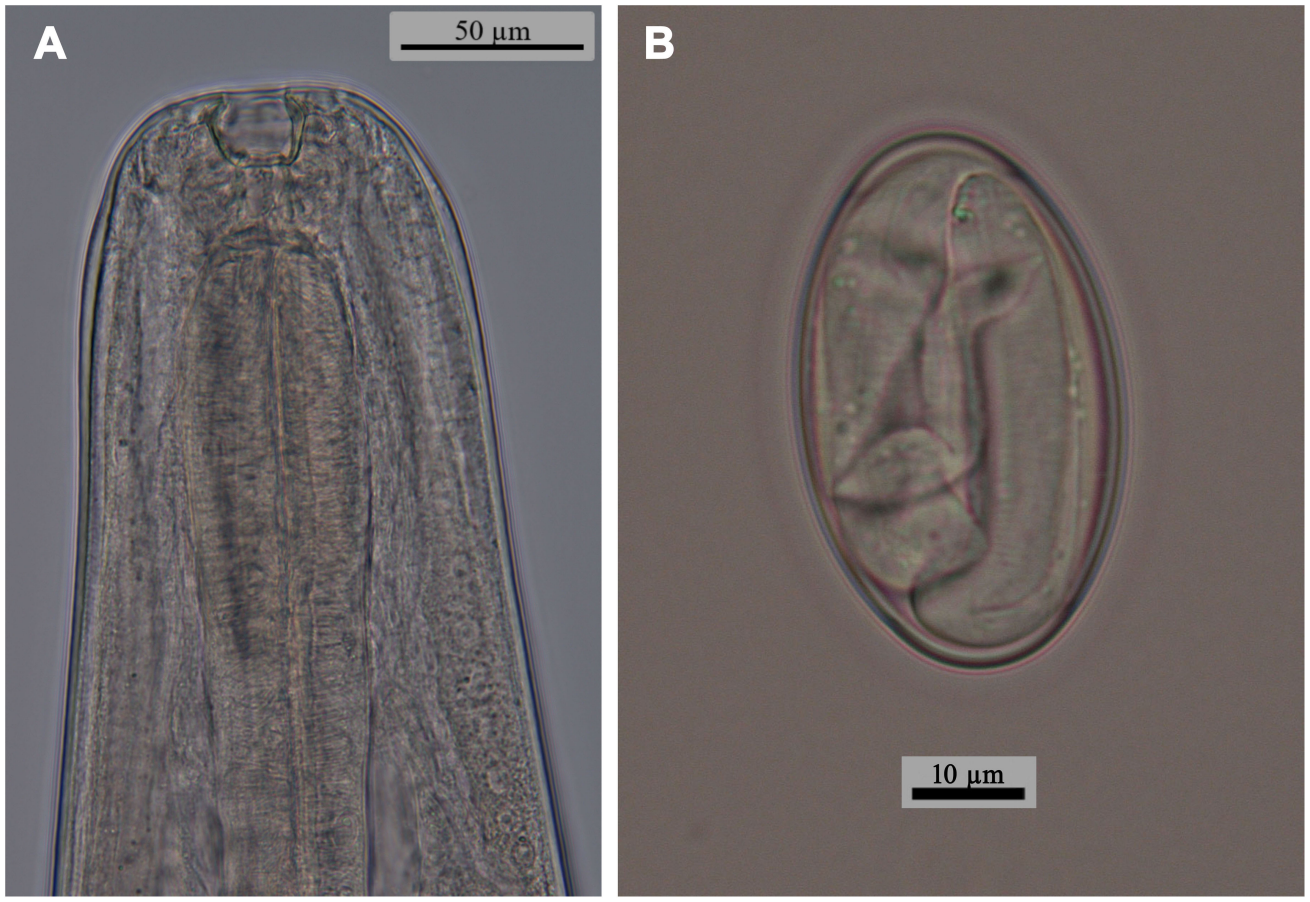
*Oxyspirura* (*Oxyspirura*) *tamarina* sp. nov. from the cotton-top tamarin *Saguinus oedipus* (Linnaeus, 1758) from a zoological garden in the Czech Republic, light microscopy micrographs. **(A)** anterior end of male, lateral view; **(B)** egg.

**Figure 3:**
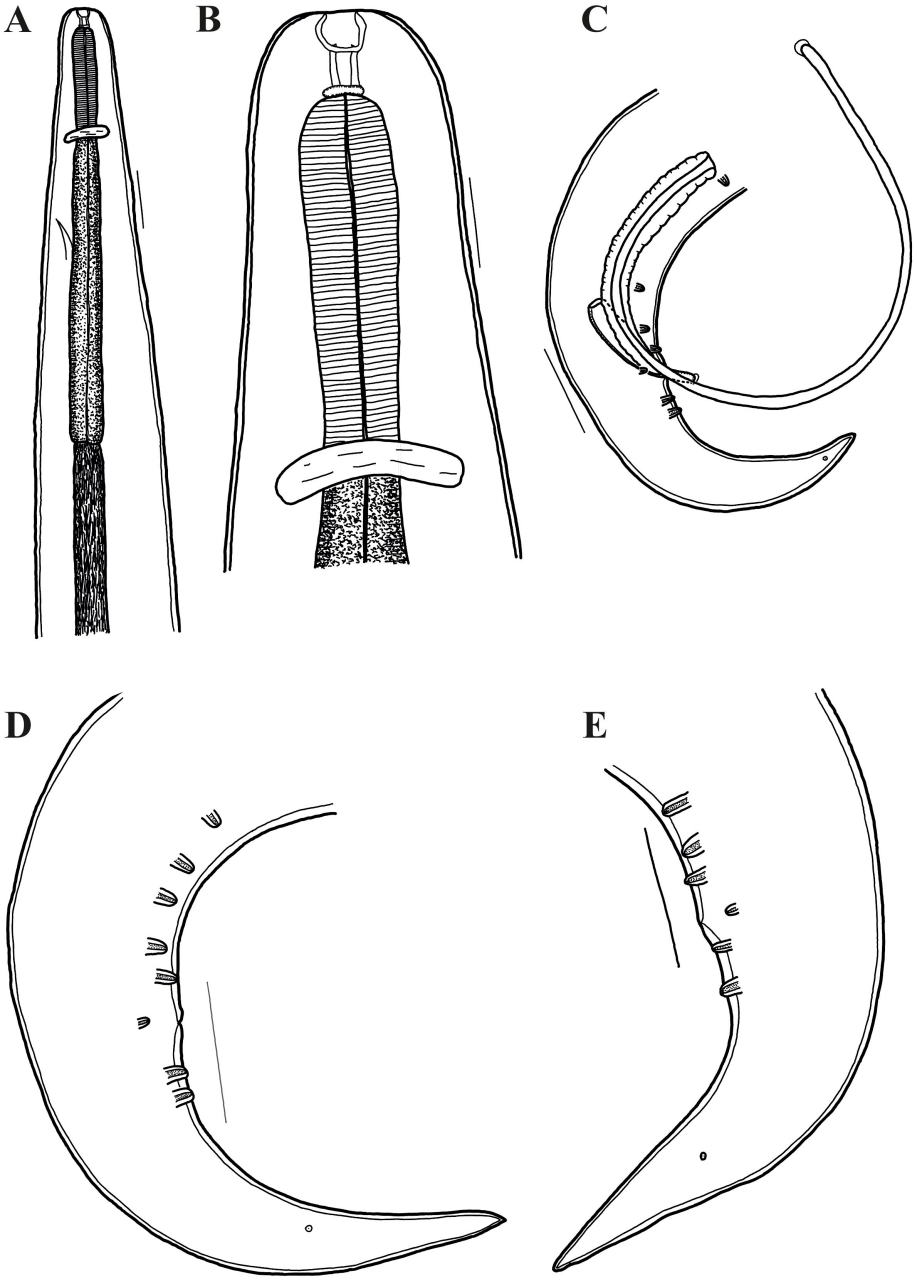
*Oxyspirura* (*Oxyspirura*) *tamarina* sp. nov. in three tamarin species from zoological garden and private facility in the Czech Republic, line drawings. **(A, B)** anterior end of male, lateral views; **(C)** posterior end of male, lateral view; **(D, E)** posterior end of male from *Saguinus oedipus*, lateral views. Scales: **A**: 100 μm, **B**: 50 μm, **C–E**: 100 μm.

**Figure 4:**
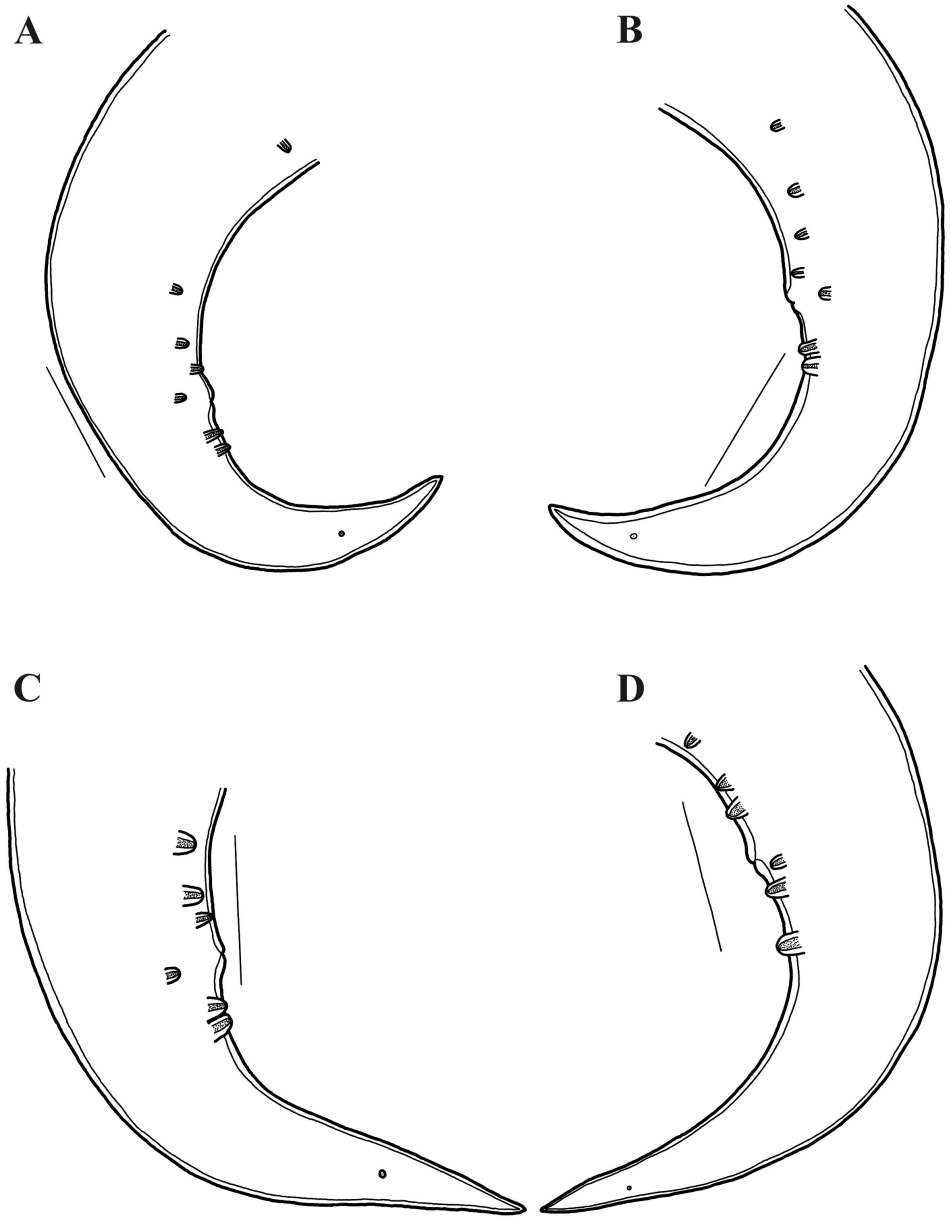
*Oxyspirura* (*Oxyspirura*) *tamarina* sp. nov. in three tamarin species from zoological garden and private facility in the Czech Republic, line drawings. **(A, B)** posterior end of male from *Leontopithecus chrysomelas*, lateral views; **(C, D)** posterior end of male from *S*. *oedipus*, lateral views (different specimen). Scales: **A–D**: 100 μm.

**Figure 5:**
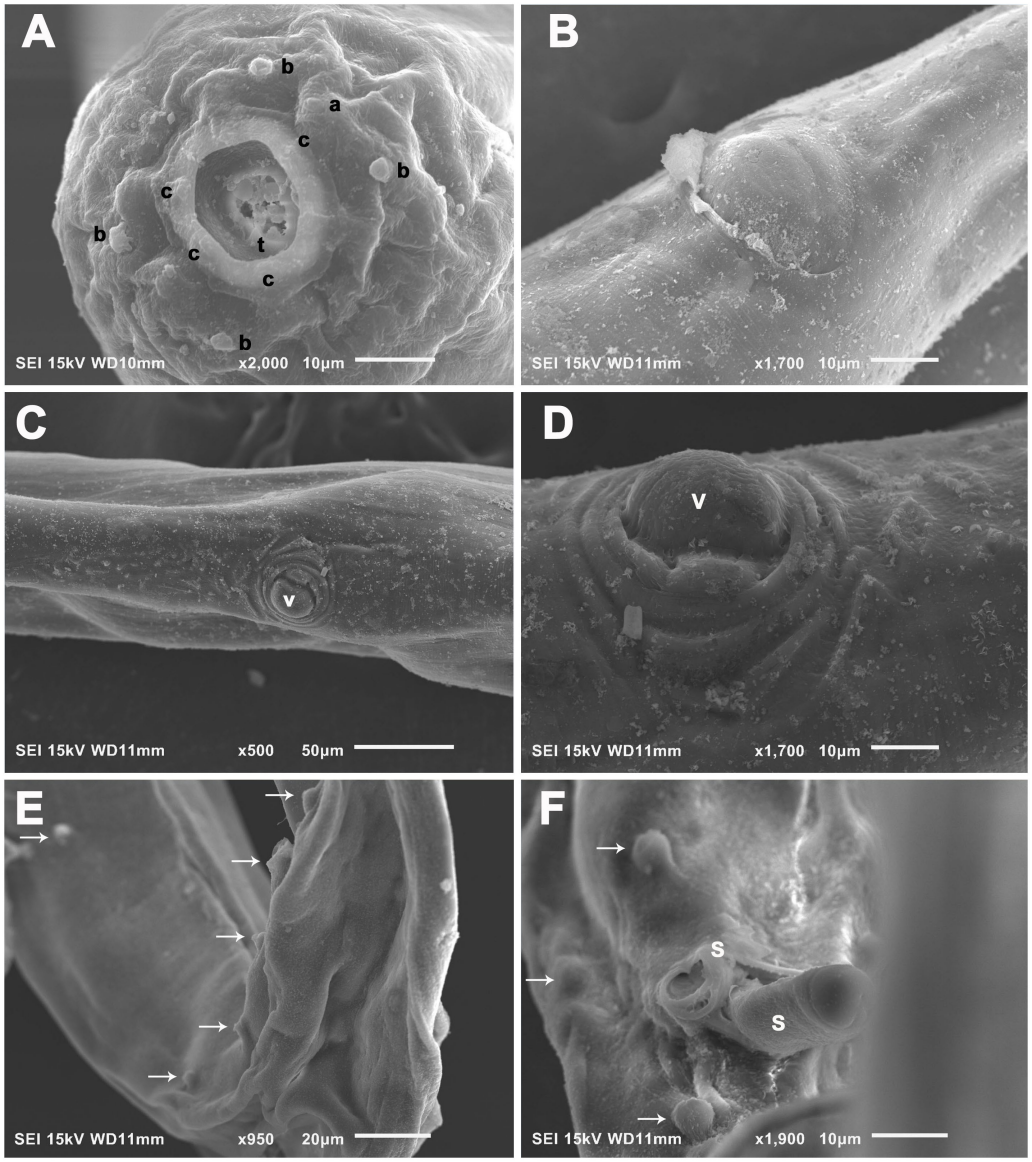
*Oxyspirura* (*Oxyspirura*) *tamarina* sp. nov. in three tamarin species from zoological gardens and private facility in the Czech Republic, electron scanning micrographs. **(A)** Cephalic end of male, apical view. **(B)** Female, region of anus, ventral view. **(C, D)** Female, region of vulva, ventral and subventral views, respectively. **(E, F)** Posterior end of male, dorsoventral and ventral views, respectively (arrows indicate caudal papillae). a, amphids; b, cephalic papillae of outer circle; c, cephalic papillae of inner circle; s, spicule; t, tooth; v, vulva.

*Oxyspirura* (*Oxyspirura*) *tamarina* sp. nov. (Figures 1–5).

Description: small, whitish nematodes. Anterior end rounded; posterior end pointed. Cuticle with longitudinal discontinuous crests, transverse annulations and thin striae. Lateral chord present. Mouth opening oval, surrounded by six papillae on inner circle (four double submedian, two single lateral), four external submedian single cephalic papillae, and a pair of lateral amphids (Figure 4A). Buccal capsule sclerotized, divided into upper and lower halves by a transverse ring; anterior half with tooth-like thickenings at its base (Figures 2A, 3A, B, 5A); posterior half funnel-shaped and smooth. Esophagus divided into muscular and glandular parts, the latter longer and broader than the former. Excretory pore posterior to nerve ring (Figure 3A). Deirids small, spike-like, situated between nerve ring and excretory pore. Tail conical.

Male (holotype from *Saguinus oedipus*, measurements of 1 specimen from *Leontopithecus chrysomelas* in parentheses, measurements of 1 specimen from *Saginus oedipus* in brackets): body length 8.45 (6.77) [7.75] mm; maximum width 250 (212) [250]. Distance of nerve ring, excretory pore and deirids 234 (198) [224], 326 (326) [326], – (–) [261], respectively, from anterior end of body. Anterior part of buccal capsule 9 (9) [9] long, 22 (19) [10] wide; its posterior part 17 (19) [11] long and 12 (9) [12] wide. Length of entire esophagus 775 (663) [714]. Testes reaching anteriorly last third of esophagus. Spicules unequal, dissimilar; left spicule 1,372 (886) [1,295] long, slender, with short handle and very long lamina, distal end with membranous structure; right spicule short, 199 (141) [159] long, with proximal end broad and distal end rounded bearing membranous distal extremity (Figures 3C, 5F). Gubernaculum 32 (absent) [47] long (Figures 1B, C). Caudal papillae: 5 (4) [3] subventral precloacal pairs on right side, 3 (4) [3] on left side; 1 (1) [1] subventral adcloacal pair, 2 (2) [2] subventral postcloacal pairs (Figures 4A–D, 5E, F). Postlcloacal pairs separated from each other on left side, close together on right side of holotype (Figures 3D, E). Phasmids small, lateral, at posterior third of tail. Tail bent in ventral direction, 357 (306) [367] long (Figures 1B, 3C).

Gravid female (allotype from *S*. *oedipus*, measurements of 4 specimens from *Leontopithecus chrysomelas* in parentheses, measurements of 4 specimens from *Saginus oedipus* in brackets): Body length 10.45 (8.57–10.57) [9.75–11.80] mm; maximum width 325 (237–300) [300–325]. Distance of nerve ring, excretory pore and deirids 204 (193–239) [255], 346 (275–387) [336–377], – (244–285) [–], respectively, from anterior end of body. Anterior part of buccal capsule – (12–14) [9–13] long, – (19–24) [19–26] wide; its posterior part 19 (19) [19–27] long and 14 (14) [7–13] wide. Length of entire esophagus 795 (714–754) [724–826]. Vulva slit-like, slightly anterior to anus, 9.75 (7.75–9.87) [8.95–10.87] mm, from anterior body end, with protruded lips, surrounded by numerous elongate folds, often extended more lateral and posteriorly than anteriorly to vulva, as well as numerous bosses posterior to vulva and absent anteriorly to it (Figures 5C, D). Vagina directed anteriorly. Amphidelphic, uterus reaching anteriorly to level of mid esophagus and posteriorly to level of anus. Larvated eggs oval, with thin and smooth shell (Figures 1D, E, 2B), 42–47 (39–44) [34–44] long and 24–27 (27–29) [19–27] wide. Larvae elongate, 187–190 long (Figure 1F). Tail length 265 (285–346) [295–316]. Anus opening slit-like, with posterior lip slightly protruded (Figure 5B).

Young female (based on 2 specimens from *Saginus oedipus*): body length 7.65–7.95 mm; maximum width 200–237. Distance of nerve ring, excretory pore, and deirids 193–224, 244–326, and 236–285, respectively, from anterior end of body. Anterior part of buccal capsule 12 long, 19–22 wide; its posterior part 19 long and 14 wide. Length of entire esophagus 714–734. Vulva slit-like, slightly anterior to anus, 6.77–7.22 mm from anterior body end, with protruded lips, surrounded by numerous large cuticular bosses, often extended more posteriorly than anteriorly to vulva. Vagina directed anteriorly. Poorly developed eggs oval, with thin and smooth shell. Tail length 204–275.

Type host: Cotton-top tamarin *Saginus oedipus* (Linnaeus, 1758) (Mammalia, Primates).

Other hosts: Golden-headed lion tamarin *Leontopithecus chrysomelas* (Kuhl, 1820), Midas tamarin *Saguinus midas* Linnaeus, 1758 (Mammalia, Primates).

Site of infection: Both eyes.

Type locality: Zoological Garden at Czech Republic.

Other locality: Private breeder facilities at Czech Republic.

Deposition material: Helminthological collection. Male holotype accession number (ECOPA−136H), female allotype accession number (ECOPA−136A), paratypes accession numbers (ECOPA−136) (male and females). All sequence data generated were deposited in the GenBank database: *18s* rRNA (PV661113), *28S* rRNA (PV661114), *cox1* (PV655167), ITS (PV704038).

ZooBank registration: To comply with the regulations set out in article 8.5 of the amended 2012 version of the International Code of Zoological Nomenclature (ICZN) (9), details of the new species have been submitted to ZooBank. The Life Science Identifier (LSID) for *Oxyspirura* (*O*.) *tamarina* sp. nov. is urn: lsid:zoobank.org:pub:8C828578-DE7A-4A59-8037-E3E7D2DA25B1.

Etymology: The species epithet is due to the French name given to the New World non-human primates (i.e., tamarin).

Remarks: The genus *Oxyspirura* is primarily divided in six subgenera according to the presence of an undivided (i.e., *Barusispirura* Chabaud, 1975, *Cramispirura* Skrjabin, 1931, *Hamulofilaria* Chandler, 1924 [= *Skrjabinispirura* Baruš, 1963], *Molinospirura* Rodrigues, 1986) or a divided buccal capsule (i.e., *Caballeroispirura* Baruš, 1963, *Oxyspirura* [= *Yorkeispirura*] Skrjabin, 1931). The nematodes examined were identified as belonging to the subgenus *Oxyspirura* due mainly to the presence of a divided buccal capsule, absence of lateral alae, and unequal and dissimilar spicules [see (10)]. Therefore, the new species can be easily distinguished from the species included in the subgenera *Barusispirura, Cramispirura, Hamulofilaria*, and *Molinospirura* by having a divided buccal capsule (vs. undivided), as well as from the subgenus *Caballeroispirura* by the absence of the lateral alae (vs. lateral alae present).

Out of those species within the subgenus *Oxyspirura*, the new species differs from *O*. (*O*.) *acuticauda* Jairapuri et Siddiqi, 1967, *O*. (*O*.) *apapillata* Guerrero, 1971, *O*. (*O*.) *cisticola* Sultana, 1964, *O*. (*O*.) *dicruri* Jairapuri et Siddiqi, 1967, *O*. (*O*.) *solitaria* Jairapuri et Siddiqi, 1967, *O*. (*O*.) *sturnia* Jairapuri et Siddiqi, 1967, and *O*. (*O*.) *turcottei* Addison, 1978, by having larger left spicules (886–1,372 μm vs. range of all these species 230–615 μm). *Oxyspirura* (*O*.) *alauda* Ali, 1960, *O*. (*O*.) *alii* Sultana, 1964, *O*. (*O*.) *buccosulcata* Singh, 1948, *O*. (*O*.) *dicruricola* Jairapuri et Siddiqi, 1967, *O*. (*O*.) *dukhunensis* Sultana, 1964, *O*. (*O*.) *egretta* Sultana, 1964, *O*. (*O*.) *eremopterixa* Sultana, 1964, *O*. (*O*.) *grandipapillata* Jairapuri et Siddiqi, 1967, *O*. (*O*.) *hyderabadensis* Rasheed, 1960, *O*. (*O*.) *kaitingensis* Hsü, 1933, *O*. (*O*.) *laharpurensis* Jairapuri et Siddiqi, 1967, *O*. (*O*.) *leiperi* Sultana, 1964, *O*. (*O*.) *malabarica* Jairapuri et Siddiqi, 1967, *O*. (*O*.) *meropsicola* Jairapuri et Siddiqi, 1967, *O*. (*O*.) *nigerica* Jairapuri et Siddiqi, 1967, *O*. (*O*.) *orientalis* Jairapuri et Siddiqi, 1967, *O*. (*O*.) *otocompsa* Rasheed, 1960, *O*. (*O*.) *prinia* Ali, 1960, *O*. (*O*.) *rustica* Jairapuri et Siddiqi, 1967, *O*. (*O*.) *singhi* Rasheed, 1960, *O*. (*O*.) *suraiyae* Sultana, 1965, and *O*. (*O*.) *wellsi* Jairapuri et Siddiqi, 1967 differ from *O*. (*O*.) *tamarina* sp. nov. in the shorter left and right spicules (range of all these species, right 66–170, left 140–420 μm vs. right 141–199, left 886–1,372 μm). Meanwhile, *O*. (*O*.) *basiri* Siddiqi et Jairapuri, 1964, *O*. (*O*.) *brasiliensis* Rodrigues, 1962, *O*. (*O*.) *cameroni* Strachan, 1957, *O*. (*O*.) *chabaudi* Baruš, 1965, *O*. (*O*.) *cruzi* Rodrigues, 1962, *O*. (*O*.) *fulica* Sultana, 1964, *O*. (*O*.) *lobipluvia* Ali, 1960, *O*. (*O*.) *lumsdeni* Addison et Anderson, 1969, *O*. (*O*.) *matogrosensis* Rodrigues, 1963, *O*. (*O*.) *mirzai* Jairapuri et Siddiqi, 1967, *O*. (*O*.) *petrowi* (Skrjabin, 1929) Skrjabin, 1931, *O*. (*O*.) *pici* Borgarenko, 1984, *O*. (*O*.) *sygmoidea* (Molin, 1860) Cram, 1937, and *O*. (*O*.) *tanasijtchuki* (Skrjabin, 1916) Skrjabin, 1931, have larger or similar length of right spicules, but smaller left spicules (range of all these species, right 121–320, left 250–760 μm).

*Oxyspirura* (*O*.) *chauvancyi* Díaz-Ungria, 1963, *O*. (*O*.) *diazungriai* Guerrero, 1969, *O*. (*O*.) *guriensis* Guerrero, 1969, *O*. (*O*.) *hispanica* Yeh, 1957, *O*. (*O*.) *mansoni* (Cobbold, 1879) Skrjabin, 1931 have similar length of the right spicule, but larger left spicule (range of all these species, right 143–410 μm, left 2.34–5.70 mm) than the new species. Particularly, *O*. (*O*.) *pusillae* Wehr et Hwang, 1957 has similar length of the right spicule, but larger left spicules (right 149–242 μm, left 1.70–2.40 mm).

Morphologically, the present nematodes are more similar in the body length of males (6.77–8.45 vs. 5.00–7.00 and 6.30–9.40 mm, respectively), females (8.57–11.80 vs. 8.00–11.00 and 10.30–12.60 mm, respectively), length of spicules (left 886–1,372, right 141–199 μm vs. left 1,40 and 1.12–1.40, right 168 and 145–195 μm, respectively), and number and distribution of ad- and postcloacal papillae (1 adcloacal, 2 postcloacal) to the only two species of the genus reported in non-avian hosts, *O*. (*O*.) *conjunctivialis* and *O*. (*O*.) *youngi*. However, some specimens of the new species differ from them in the number and distribution of bosses in the vulvar region, number of precloacal papillae, as well as in the absence of gubernaculum and unpaired ventral precloacal papilla.

**Figure 6:**
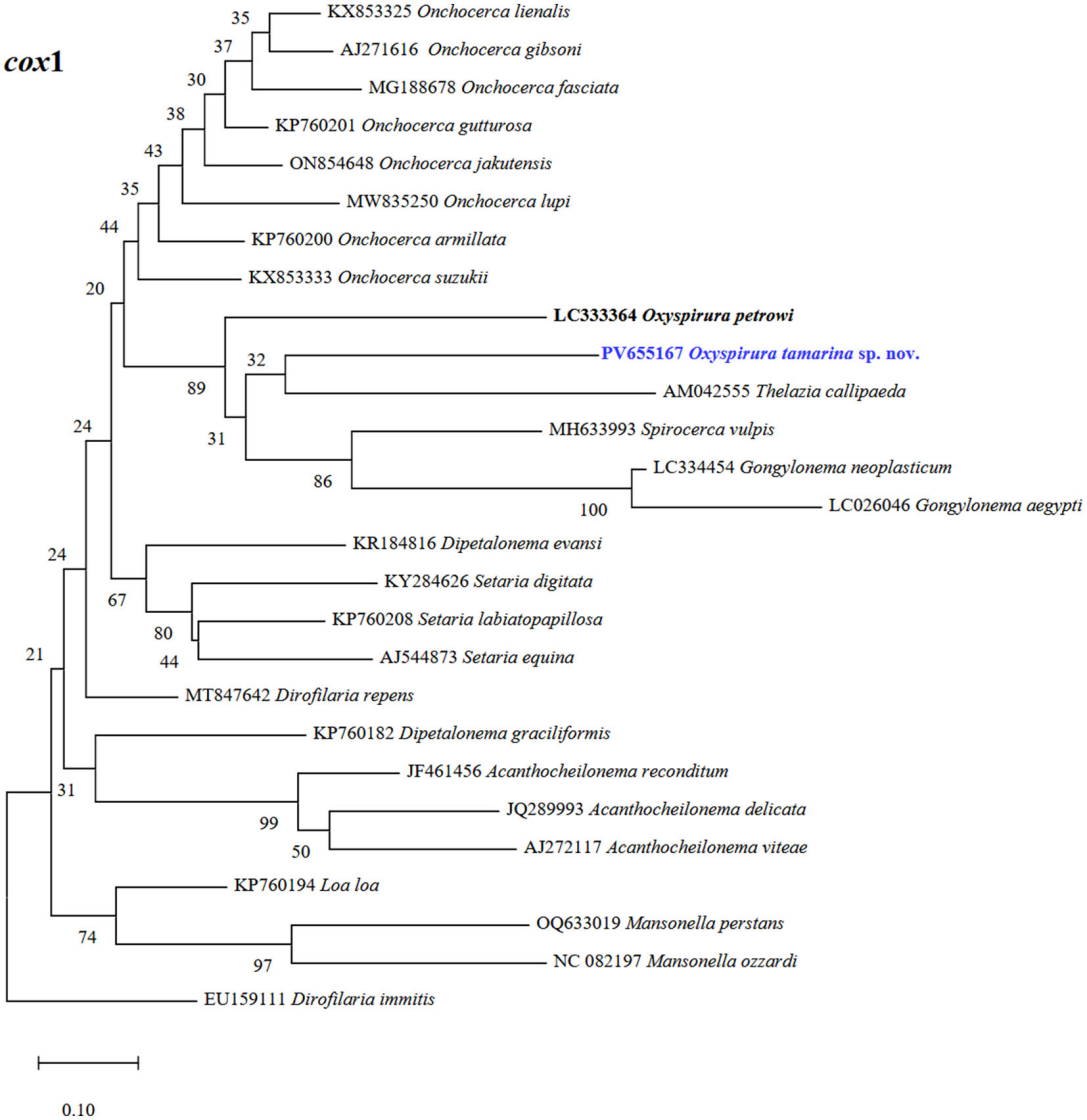
Phylogenetic tree of *Oxyspirura* (*Oxyspirura*) *tamarina* sp. nov. and its related spirurid species, based on *cox1* sequences. Highlighted blue name represent the sequence obtained in the present study, while those in bold are congeneric species. The numbers on phylogenetic trees are bootstrap values based on 1,000 replications, while those before each nematode species are GenBank accession numbers.

**Figure 7:**
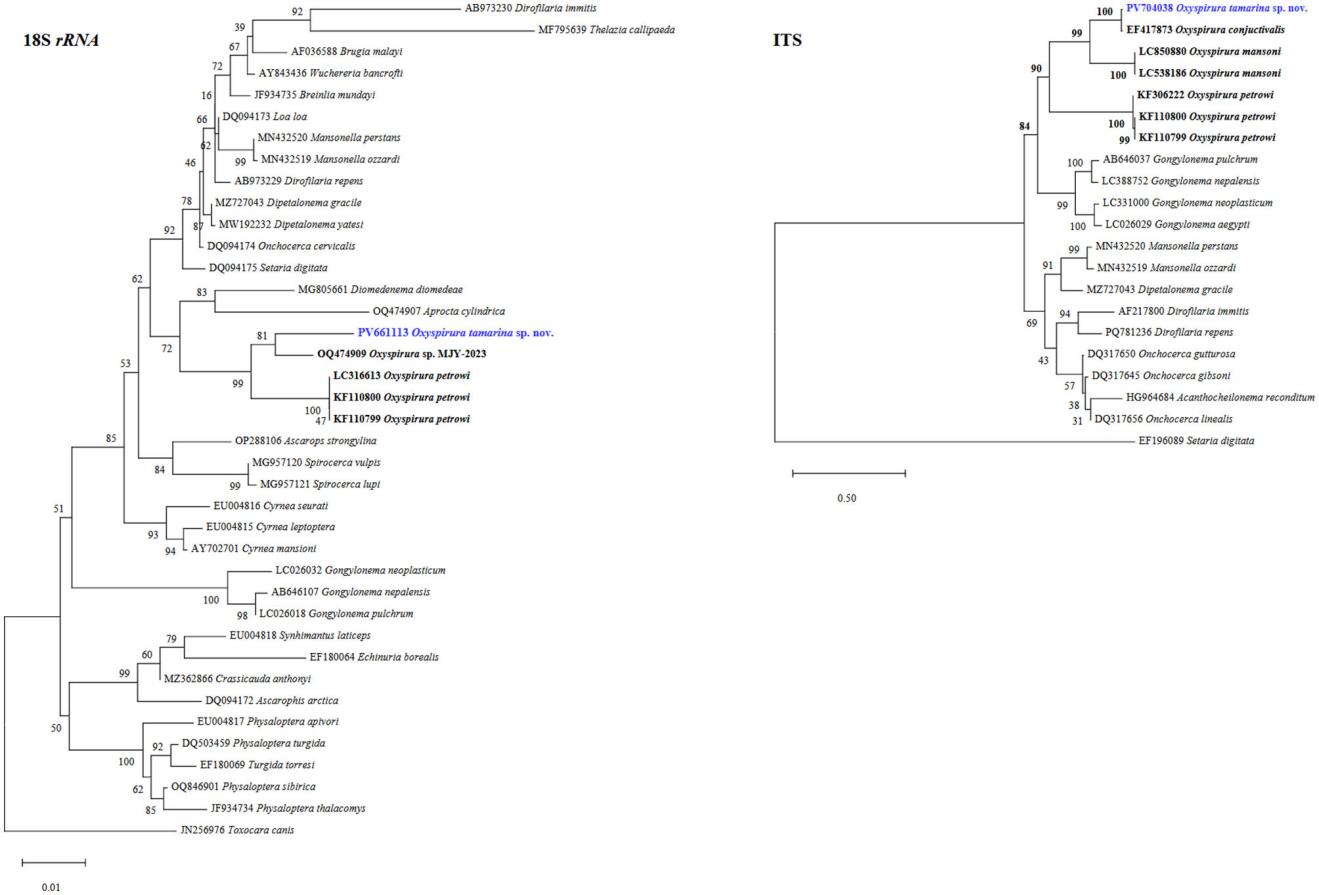
Phylogenetic trees of *Oxyspirura* (*Oxyspirura*) *tamarina* sp. nov. and its related spirurid species, based on *18S* rRNA, and ITS region sequences. Highlighted blue names represent the sequences obtained in the present study, while those in bold are congeneric species. The numbers on phylogenetic trees are bootstrap values based on 1,000 replications, while those before each nematode species are GenBank accession numbers.

Molecularly, all the new generated sequences were identical to each other. At *cox1* gene, the sequence of *O*. (*O*.) *tamarina* sp. nov. (PV655167, 607 bp) was similar to that of *Onchocerca gutturosa* (Neumann, 1910) (KP760201; 632 bp) in *Bos taurus* Linnaeus, 1758 from Cameroon, and *On*. *linealis* Johnston, 1921 (KX853326; 632 bp) in *B*. *taurus* from Wales (88.63, 88.47%, respectively). *Dirofilaria repens* Railliet et Henry, 1911 (MT847642; 678 bp) in *Homo sapiens* Linnaeus, 1758 from Croatia, *O*. *petrowi* (PV203592; 661 bp) in *Buteo buteo* (Linnaeus, 1758) from Romania, and *O*. *petrowi* (LC333364; 598 bp) in *Colinus virginianus* (Linnaeus, 1758) from Texas were less similar (87.83, 87.23, 85.04%, respectively), as well as to those of *O*. *mansoni* (LC538191, LC850883; 393 bp) in the eyes of domestic chickens in Bangladesh and Viet Nam (both 88.89%), although they have only 28% of query cover. In the phylogenetic tree, the new species forms a cluster along with a congeneric species (*O*. *petrowi* LC333364), with a nematode species found in the eyes (*Thelazia callipaeda* AM042555; 689 bp; 85.57% similar) or upper digestive tract (*Gongylonema aegypti* LC026046; 841 bp, *G*. *neoplasticum* LC334454; 852 bp; 83.20 and 83.36% similar, respectively) of birds and mammals, as well as *Spirocerca vulpis* (MH633993; 846 bp; 85.17% similar) in the red fox (Figure 4). At ITS (the partial *18S*, complete ITS1–*5.8S*–ITS2, and partial *28S* sequences) (PV704038; 1410 bp), the new species showed 94.52% similarity to that of *O*. (*O*.) *conjunctivialis* (EF417873; 689 bp) in *N*. *c*. *coucang* from Russia, and 74.77% to *O*. *mansoni* (LC538186; 1189 bp) in domestic chickens from Bangladesh; and it forms a larger clade formed by three subclades with other *Oxyspirura* species (Figure 6). At *18S* rRNA gene our sequence (PV661113; 1724 bp) formed a cluster along with the sequence of one unnamed *Oxyspirura* MJY-2023 (OQ474909; 1727 bp) in *Strix varia* Barton, 1799 from California (97.90% of similarity), as well as with three sequences of *O*. *petrowi* (KF110799; 2715 bp, KF110800; 2698 bp, LC316613; 1811 bp) found in *Colinus virginianus* from Texas (97.06% of similarity) (see Figure 7). At *28S* rRNA, the partial sequence of *O*. (*O*.) *tamarina* sp. nov. (PV661114; 683 bp) is most similar to *Dipetalonema gracile* (Rudolphi, 1809) (MZ727043; 6426 bp) in the abdominal cavity of *Saguinus bicolor* Spix, 1823, and *Mansonella ozzardi* Manson, 1897 (MN432519; 6976 bp) in *Homo sapiens* both from Brazil (93.42, 93.13%, respectively).

## Discussion

After the examination of the different non-human primate types (capuchin, macaque, marmoset, squirrel, and tamarin) and considering their similar feeding habits (based mostly in fruits, flowers and insects) [see (11)], it is surprising that only three tamarin species showed infections in eyes with *O*. (*O*.) *tamarina* sp. nov. Since the animals were kept under different captive conditions and no samples of the insects cohabiting around the enclosures were collected, the way of infection is unknown and apparently there is a certain degree of susceptibility and vulnerability among individual non-human primates or the availability of intermediate hosts might vary in the various facilities, but we do not know the extent of such variation. Several kinds of insects (e.g., cockroaches) coexist with the caged animals (breeders, personal communication), so the trophic interaction between them should be common and thus facilitate the transmission of parasites.

The morphological features of the nematodes in the tamarin species suggested that they might belong to different species, but the molecular analysis allowed us to recognize them as a sole species, thus becoming the third species of *Oxyspirura* reported in primates kept in captivity around the world. The new species showed some identical characters to the specimens originally revised and identified as *O*. (*O*.) *conjunctivialis* and *O*. (*O*.) *youngi* [see (4–6)] and might be conspecific. However, these two *Oxyspirura* species were synonymized based only on their morphology (6), while the molecular comparison lacked because just the nematodes of *N*. *c*. *coucang* were sequenced. Therefore, and based on the anatomical and genetic differences, we considered the present nematodes as a new species, although their taxonomic status might change or not after all nematodes from the eyes of captive mammals are sequenced and compared.

An interesting feature of the new species is the intraspecific variability in the length of the spicules, presence/absence of gubernaculum, and in the number and distribution of the caudal papillae. This fact has been shown in other species of *Oxyspirura*, such as *O*. (*O*.) *youngi* (now considered as a junior synonym of *O*. (*O*.) *conjunctivialis*, see (6)), and in *O*. (*O*.) *turcottei* [see (5, 12)]. The reason for such variation is still uncertain and might be related to the host species but requires a revision of the already described type species and an integral study involving morphological and molecular approaches. In this regard, the results showed that morphological characters commonly used as diagnostic features (i.e., presence/absence of gubernaculum, distribution and number of caudal papillae), at least for *Oxyspirura* in captive mammals, are apparently doubtful. The gubernaculum was absent in nematodes of *L*. *chrysomelas* and present in *S*. *midas* and *S*. *oedipus*, while the number of precloacal papillae was 4 on right side/4 in left side in *L*. *chrysomelas*, 3/3 in *S*. *midas*, and 5/4 and 3/3 in *S*. *oedipus*, although molecularly they were identical. Since members of *Oxyspirura* are more commonly found in birds, the intraspecific variability should be analyzed in the known and in those species being recently incorporated into the genus, because such variability was already recorded in *O*. (*O*.) *turcottei* in *Meleagris gallopavo silvestris* from West Virginia (USA) (12).

This is the first time that four genetic markers are used to sequence an *Oxyspirura* species. The full intraspecific similarity of the new sequences at each single marker confirms that they represent the same species, whose interspecific similarities were higher at *18S* rRNA and *28S* rRNA genes (around 97%, and 93%, respectively), while at *cox1* (87–89%) and ITS (74–94%) were less similar. Despite the higher similarities at the nuclear genes *18S* rRNA and *28S* rRNA, these markers show low species resolution and fail in separating closely related species [see (13, 14)], so they were not resolutive in our case. On the other hand, *cox1* gene and ITS region have better results in the distinction of species [see (14)], although the former seems to be the most suitable marker for the high number of sequences in GenBank (14) and because it has been recognized as an appropriate tool for the identification of nematodes [see (15)]. The higher degree of sequence variation of *cox1* gene allowed to determine the relationships between closely related species and it has been widely used for molecular identification at species and population level, as well as to differentiate helminths from various hosts species (13). Sequencing of the four markers from nematodes generated a most comprehensive genetic information for future studies, as already stated by Mejías-Alpízar et al. (14).

Apparently, infections in captive non-human primates represent incidental events caused by a favored and intense transmission in zoos or private facilities, thus leading to a more extensive infection in non-natural hosts, as already stated by Ivanova et al. (6). As above mentioned, the vast majority of the known species of *Oxyspirura* have been reported in avian hosts and are apparently host specific, since each host has its own nematode species (16). However, the occurrence of the new *Oxyspirura* species in non-human primates supports the fact that these bird nematodes are also capable of maturing in mammals, although in an incidental way because they are probably acquired from other animals while kept in, for example, zoological gardens. How the non-human primates got infected with the nematodes is uncertain, but as in other *Oxyspirura* species, these primates might acquire the infection through eating insects (e.g., burrowing cockroaches, grasshoppers, crickets) which might act as intermediate hosts (1, 6, 17, 18).

From the diagnostic point of view and considering that *Oxyspirura* nematodes are site-specific to eyes, the first detecting method of eggs should be through eye smears, although when eggs are swollen along with lacrimal secretions, they might be detected by fecal smears as reported by Kalyanasundaram et al. (2). Thus, eye smears are also a reliable method to detect the presence of eggs of the nematode in the host. Additionally, and after detecting the eggs, first-stage larvae can be characterized after manually releasing them from eggs and see the conical and pointed tail, without papilla-like protuberances. Fielding (19) and Schwabe (20) reported the presence of “four small papillae” on tail of only the second-stage larvae of *O*. *mansoni*, although Ivanova et al. (6) mentioned that they likely belong to another spirurid nematode due to the presence of such digitiform structures. Apparently, these “papillae” only occur in the second-stage larvae, while they are absent in the remaining stages (unhatched, first-stage and fourth-stage larva), so their presence/absence should always be determined to avoid misidentifications.

Since most of the studied non-human primates are handled and raised by humans, there is certain risk in the development of a possible zoonotic infection by the eye worms. Recently, Doanh et al. (3) found that the larvae of *O*. *mansoni* in domestic chickens from Vietnam are able to infect humans and cause dermatitis with visible larva migrans under the skin of various body parts. Even though the way of infection is unclear, these authors mentioned that larvae released from dead or injured cockroaches could penetrate human skin when individuals walk barefoot in chicken coops or after eating grasshoppers, but these hypotheses still need to be solved (3). Therefore, sanitary procedures should be followed while breeding these non-human primates.

An integrative analysis, by using traditional and molecular approaches, was applied to uncover the specific identity of the nematodes found in the three tamarin species. The results clearly showed that despite the morphological variability of the specimens, they all belong to the same species. Molecular analyses are shown as a powerful tool for taxonomy, but without the integration of traditional morphological studies it might become a simple collection of molecular taxonomic units (morphospecies), thus suggesting that an integrated approach to species recognition is always needed.

## Data Availability

The datasets presented in this study can be found in online repositories. The names of the repository/repositories and accession number(s) can be found in the article/supplementary material.
